# It's not too Late for the Harpy Eagle (*Harpia harpyja*): High Levels Of Genetic Diversity and Differentiation Can Fuel Conservation Programs

**DOI:** 10.1371/journal.pone.0007336

**Published:** 2009-10-05

**Authors:** Heather R. L. Lerner, Jeff A. Johnson, Alec R. Lindsay, Lloyd F. Kiff, David P. Mindell

**Affiliations:** 1 Smithsonian Institution, National Zoological Park, Genetics Lab, Washington, DC, United States of America; 2 University of Michigan, Museum of Zoology and Department of Ecology and Evolutionary Biology, Ann Arbor, Michigan, United States of America; 3 Department of Biological Sciences, Institute of Applied Sciences, University of North Texas, Denton, Texas, United States of America; 4 Department of Biology, Northern Michigan University, Marquette, Michigan, United States of America; 5 The Peregrine Fund, Boise, Idaho, United States of America; 6 California Academy of Sciences, San Francisco, California, United States of America; University of Uppsala, Sweden

## Abstract

**Background:**

The harpy eagle (*Harpia harpyja*) is the largest Neotropical bird of prey and is threatened by human persecution and habitat loss and fragmentation. Current conservation strategies include local education, captive rearing and reintroduction, and protection or creation of trans-national habitat blocks and corridors. Baseline genetic data prior to reintroduction of captive-bred stock is essential for guiding such efforts but has not been gathered previously.

**Methodology/Findings:**

We assessed levels of genetic diversity, population structure and demographic history for harpy eagles using samples collected throughout a large portion of their geographic distribution in Central America (n = 32) and South America (n = 31). Based on 417 bp of mitochondrial control region sequence data, relatively high levels of haplotype and nucleotide diversity were estimated for both Central and South America, although haplotype diversity was significantly higher for South America. Historical restriction of gene flow across the Andes (i.e. between our Central and South American subgroups) is supported by coalescent analyses, the haplotype network and significant *F*_ST_ values, however reciprocally monophyletic lineages do not correspond to geographical locations in maximum likelihood analyses. A sudden population expansion for South America is indicated by a mismatch distribution analysis, and further supported by significant (*p*<0.05) negative values of Fu and Li's *D_F_* and *F*, and Fu's *F*_S_. This expansion, estimated at approximately 60 000 years BP (99 000–36 000 years BP 95% CI), encompasses a transition from a warm and dry time period prior to 50 000 years BP to an interval of maximum precipitation (50 000–36 000 years BP). Notably, this time period precedes the climatic and habitat changes associated with the last glacial maximum. In contrast, a multimodal distribution of haplotypes was observed for Central America suggesting either population equilibrium or a recent decline.

**Significance:**

High levels of mitochondrial genetic diversity in combination with genetic differentiation among subgroups within regions and between regions highlight the importance of local population conservation in order to preserve maximal levels of genetic diversity in this species. Evidence of historically restricted female-mediated gene flow is an important consideration for captive-breeding programs.

## Introduction

**Figure 1:**
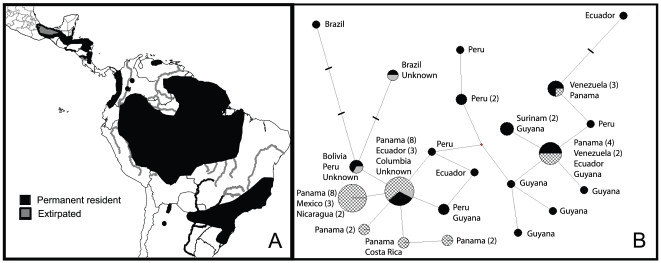
Harpy eagle geographic distribution (A) and haplotype network (B). Median-joining network (B) depicting relationships among control region haplotypes from harpy eagles sampled in South America (black), Central America (hatched) and three individuals of unknown origin (grey). Circle size is proportional to the number of individuals with that haplotype. Small gray circles at nodes and dash marks along branches indicate nucleotide substitutions required to connect sampled haplotypes.

Harpy eagles (*Harpia harpyja*) are the largest extant birds of prey in the New World with females, the larger sex, weighing as much as 9.0 kg. They feed on sloths, monkeys and other arboreal mammals as well as large birds, such as guans, curassows, and macaws of lowland rainforests [1]–[6]. The harpy eagle is the only extant member of its genus and although it is similar in plumage to its closest known living relative, the crested eagle (*Morphnus guianensis*), the two are highly divergent genetically [7]. Their current distribution extends from southern Mexico to east-central Brazil Figure 1. [8], [9].

With two or more years between reproductive attempts, they may have the longest breeding period of any raptor [2], [5], [10]. Due in large part to persecution by humans [11], but also their relatively slow rates of reproduction and utilization of high-quality rainforest habitat [2], [12]–[15], the harpy eagle is a species of conservation concern. A pattern of habitat degradation followed by new human occupation of harpy eagle habitat and increased human persecution (i.e. shooting) leads to increasingly high loss of harpy individuals in degraded habitat where they might otherwise persist [5], [15]. The species is considered near-threatened by the World Conservation Union [16] and is listed in Appendix I (endangered species) of the Convention on International Trade of Endangered Species of Wild Fauna and Flora (CITES). Local extinctions have occurred and some remnant populations are known to be small and isolated such that they are considered endangered in several Neotropical countries [10].

Fragmentation of rainforest habitat, particularly extensive in Central America [17], has likely contributed to local extinctions of many Neotropical predators including harpy eagles [10], [18], jaguars (*Panthera onca*), ocelots (*Leopardus pardalis*) and margays (*L. wiedii*; [12], [13]) which are all thought to require large contiguous rainforest habitat not only for healthy prey populations but also for relatively large home ranges [3], [5]. Predators play a crucial role in maintaining trophic interactions of terrestrial ecosystems [e.g. 19], [20]–[23] such that their removal from established habitats is associated with drastic alterations in ecosystem dynamics, including loss of plant species diversity and population explosions of primary consumers [e.g. Lago Guri], [ Venezuela], [24], [25]–[27] Thus, studies investigating predator populations can also provide clues to the health of ecosystems [28], [29], [but see also 30].

For harpy eagles, little or no information exists on historical demography, population connectivity between geographic regions and the extent to which habitat fragmentation has affected levels of genetic diversity. High levels of natal philopatry in harpy eagles [5] may be associated with phylogeographic structure and/or inbreeding in isolated patches of habitat. Since genetic diversity is important for the persistence of populations [31]–[33], estimates of genetic variability and demographic parameters for species threatened with extinction are valuable for their conservation [34]. In particular, fitness consequences due to mitochondrial sequence variation are likely to be pronounced due to the smaller effective population size of mitochondrial versus nuclear DNA [reviewed in 35], [36].

Captive-breeding programs for harpy eagles have been undertaken in several countries with release of captive-bred individuals beginning in 1998 in Panama and 2003 in Belize [5], [37]. Baseline genetic data of pre-release populations is needed to evaluate the effect of these and any future introductions on genetic diversity levels within the wild populations. In this study we use coalescent and phylogenetic-based analyses and quantitative test statistics for mitochondrial DNA (mtDNA) control region sequence to reconstruct the population demographic history of the harpy eagle before reintroduction of captive-bred birds. In particular, we quantify levels of mitochondrial genetic diversity, assess historical levels of gene flow among geographic regions, and estimate relative maternal effective population sizes for harpy eagles from a broad geographic range across 12 countries.

## Results and Discussion

### Sequence characteristics and genetic diversity

Control region sequences of 400 to 417 bps were generated for 66 harpy eagles and a single representative of the outgroup, *Morphnus guianensis* (Supplement, Table S1). There were 32 harpy eagles sampled from Central America (i.e. areas west of the Andes, including the Darien region of Panama and western Colombia), 31 from South America and three for which the locality was unknown. Disregarding sites with ambiguities, twenty-two harpy eagle haplotypes were identified from a total of 21 variable sites, all of which were transitions (Table 1). There were seven haplotypes (four unique) and 18 haplotypes (13 unique) in Central America and South America, respectively, and three haplotypes were shared between regions. Two additional haplotypes were shared by individuals of unknown origin with individuals sampled in South America. The majority of haplotypes unique to South America were represented by only one or two individuals, with the exception of the three shared haplotypes mentioned above.

**Table 1:** Sequence characteristics from 417 bp of mitochondrial domain I control region.

Geographic region	n	variable sites/# of haplotypes	Haplotype diversity^1^, *h* ± SD	Nucleotide diversity^2^, π ± SD	*D_\_* ^3^	*D_F_* ^4^	*F* ^4^	*F_S_* ^5^
All samples^6^	66	21/23	0.906±0.020	0.00763±0.0045			–	
Central America	32	9/7	0.768±0.053	0.00518±0.0033	−0.23	1.07	0.94	−0.23
Costa Rica-Nicaragua-Mexico	6	2/2	0.333±0.220	0.00167±0.0017				0.95
Panama	26	9/7	0.803±0.047	0.00567±0.0036				−0.31
South America	31	17/18	0.955±0.018	0.00823±0.0048	−0.77	−2.59**	−2.55**	−10.20**
Colombia-Ecuador-Peru	14	9/10	0.923±0.060	0.00764±0.0047				−4.51*
Venezuela-Surinam-Guyana	14	9/8	0.901±0.052	0.00599±0.0039				−2.68**
Brazil-Bolivia	6	5/3	0.733±0.160	0.00517±0.0039				1.08

1 Nei 1987.

2 Tajima 1983.

3 Tajima 1989.

4 Fu and Li 1993.

5 Fu 1997.

6 includes three samples with unknown geographic localities.

* p<0.05, **p<0.01.

The South American region possessed significantly higher haplotype diversity (0.955±0.018; *h* ± s.e.) than Central America (0.768±0.053; *t* = 3.39, *P*<0.01). Total nucleotide diversity was similar between the regions (South America, 0.008±0.005; Central America, 0.005±0.003; Table 1). Both the smaller area of rainforest habitat historically in Central America and the higher loss of habitat compared to South America could result in a smaller population size of harpy eagles in Central America and thus account for the lower haplotype diversity there. If harpy eagles are historically a South American species, satellite populations in Central America would be expected to contain fewer haplotypes than the main South American population. However, the focus on sampling within Panama may also have resulted in the recovery of fewer haplotypes in the Central American region. While the inclusion of some older samples could have inflated the overall genetic diversity measures, we think this effect was minimal as only three haplotypes were found exclusively in samples collected before 1960 (two samples from Peru and one from Brazil). It is more likely that our genetic diversity estimates for harpy eagles are conservative and further sampling both in South America (where the majority of sampled haplotypes were represented by only one or two individuals) and in additional regions of Central America could identify even more haplotypes.

### Population subdivision

Significant genetic differentiation (*F_ST_* = 0.23, p<0.001) between Central and South America reflects restriction of gene flow around the northern extreme of the Andean range as seen with other predators in Neotropical forests, including the jaguar [13], ocelot, margay [12], puma [38], and another accipitrid species, the hook-billed kite [*Chondrohierax uncinatus*, 39]. Significant *F_ST_* values among subgroups within South America and among Central and South American subgroups (Table 2) also show a pattern of geographic differentiation with most haplotypes unique to a single area, particularly in South America, and only a few haplotypes with broad distributions. Analysis of molecular variance (AMOVA) showed substantial variation among regions (10.02%) and among subgroups within regions (22.27%) with the majority of genetic variation observed within subgroups (67.71%). Although our sampling within northern Central America is not sufficient to fully evaluate the level of connectivity or isolation of more northern areas (81% of our samples originated in eastern Panama), it should be noted that there were no haplotypes unique to Central American locations outside of Panama. That is, all haplotypes sampled in Central America were found in at least one individual from Panama. Provided that additional samples could be obtained from Central America in areas other than eastern Panama, future work on harpy eagles should investigate the potential for phylogeographic structure within this geographic area. This information is important for conservation because rainforest habitat is more fragmented throughout this area and reintroduction of individuals has been undertaken here, specifically in Belize. However, very few harpy eagles currently exist in areas outside of Panama [8]–[10].

**Table 2:** Matrix of pairwise *F_ST_* values for geographic subgroups.

Geographic subgroup	Mexico-Costa Rica-Nicaragua	Panama	Colombia-Ecuador-Peru	Venezuela-Surinam-Guyana
Mexico-Costa Rica-Nicaragua				
Panama	0.0999			
Colombia-Ecuador-Peru	0.328**	0.107*		
Venezuela-Surinam-Guyana	0.608**	0.386**	0.193**	
Brazil-Bolivia	0.570**	0.298**	0.290**	0.416**

* p<0.05, **p<0.01.

For the haplotype network, the shortest tree length had 26 nucleotide substitutions. Varying settings of epsilon had no effect on the topology. The shape of the haplotype network (Figure 1b) shows that haplotypes sampled in Central American individuals cluster together (within one to two mutational steps) with the exception of two common but more distantly related haplotypes that are also found in individuals from South America. Haplotypes recovered in South American individuals are found throughout the network with no obvious clusters. The shape of the network is compatible with a scenario of restricted gene flow between Central and South America in which each region has predominantly unique haplotypes (potentially generated during isolation) and the three shared haplotypes are internal haplotypes (likely resulting from shared ancestral polymorphisms but also possibly from recent gene flow).

The ML topology (not shown) resulting from an unconstrained analysis recovered two main clades with low support (bootstrap values of 56 and 60) that did not correspond to geographical origin and a third clade comprised of three Peruvian haplotypes each represented by a single bird (bootstrap value 94). The difference in likelihood scores between the unconstrained and constrained phylogenies was not significant (p>0.10). Our finding of non-reciprocal monophyly for mtDNA lineages based on geography supports current classification of *Harpia harpyja* as a single species and suggests a recent shared history among Central and South American populations.

### Population demographic histories

**Figure 2:**
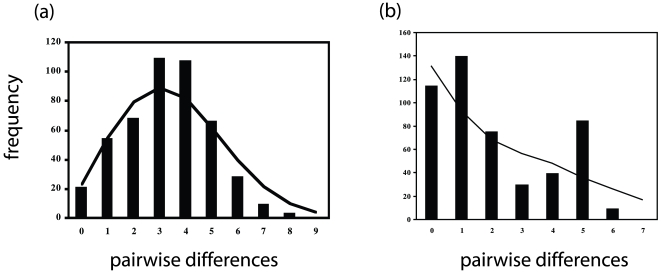
Mismatch distribution for haplotypes observed in harpy eagle samples from (a) South America and (b) Central America. The expected distribution of pairwise genetic distances among haplotypes under a model of sudden expansion are shown as a line and the observed distances are shown as vertical bars.

Within South America there was strong evidence of a recent population expansion from the shape of the mismatch distribution (SSD = 0.005, *p* = 0.063, Figure 2a), the low value of Harpending's raggedness index (*r* = 0.03, *p* = 0.084) and significant (*p*<0.05) negative values of Fu and Li's *D_F_* and *F* and Fu's *F_S_* (Table 1).

For Central America, the null hypothesis of population demographic expansion was not rejected based on the mismatch distribution (SSD = 0.023, *p* = 0.596, Fig. 2b; *r* = 0.060, *p*>0.713), but these statistics are conservative and use little information in the data [40]. Detecting population demographic size changes can be difficult with small sample sizes, few segregating sites or haplotypes, or when the population has experienced a very recent expansion [41]. Fu's *F_S_* has been shown to be more powerful than mismatch distributions in detecting demographic changes under a variety of conditions including both very recent and older population expansions [41], [42], and this statistic did not support expansion for Central America. Further, neither Fu and Li's *F* nor *D_F_* supported expansion within Central America. These two summary statistics use an outgroup sequence to identify recent intraspecific mutations and are less affected by small sample sizes than test statistics based on mismatch distributions or Fu's *F_S_* [41]. In addition, recently bottlenecked populations often show multimodal mismatch distributions with a majority of individuals with identical haplotypes [43]–[45], similar to that seen for harpy eagles in Central America where the mismatch distribution peaks between zero and two nucleotide differences and again at five nucleotide differences. Therefore, our results suggest that the Central American population may have experienced a recent bottleneck. Alternatively, recent admixture, such as gene flow from South America into Central America, could lead to a multi-modal mismatch distribution, however results from coalescent-based analyses (below) suggests that low levels of gene flow are more likely to have occurred in the reverse direction.

The estimated date of expansion calculated from τ based on the mismatch distribution for South American harpy eagles is 60 000 BP (99 000–36 000 BP 95% CI) and falls entirely within the last ice age and more specifically, well before the last glacial maximum (LGM) of 22 000–19 500 BP [46]. Following the estimated time of expansion, changes in temperature and rainfall in the Amazon basin have been associated with a decrease of rain forest and cloud forest habitat until the LGM [47] followed by expansion of these habitats to the present time. An increase in deciduous and semi-deciduous forest in the southern Amazon and grassland habitat surrounding the Amazon basin seen during the LGM is proposed to reoccur [47] coincident with current rapid global climate change involving an increase of *ca.* 3°C and a reduction of annual precipitation of ∼20% [48]. Given that harpy eagles are found only rarely in drier forests [but see 49] and population expansion for harpy eagles in South America is loosely associated with a transition to maximum precipitation in the tropical Andes (50 000–36 000 years BP [50]), anticipated climate and habitat changes present further challenges for their persistence. Alternatively, the ability of harpy eagles to persist through climatic and habitat changes both preceding and following the LGM with high levels of mitochondrial genetic diversity and without strong evidence of a population genetic bottleneck are somewhat encouraging.

### Coalescent analyses of demography

**Figure 3:**
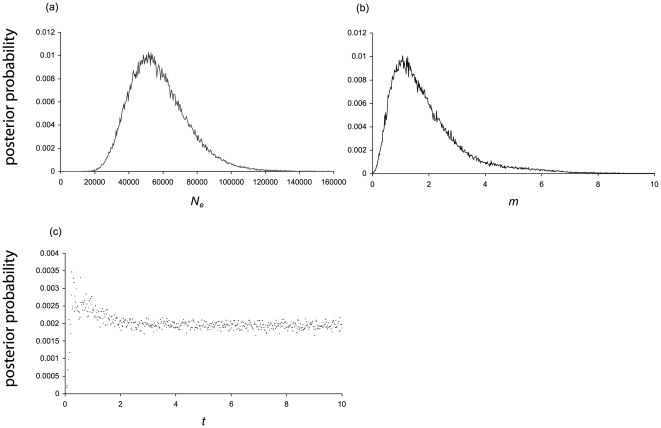
Marginal posterior probability densities from MDIV analyses. Probability densities for (a) population size, *θ*; (b) migration, *m*; and, (c) time since divergence, *t*. The x-axes correspond to the prior range of the parameters.

Given less available habitat in Central America compared to South America, it is not surprising that coalescent-based analyses in Migrate estimated a smaller long-term female effective population size (*N*_fe_) in Central America (θ_CA_ = 0.0034, 90% CI = 0.00216–0.0060; θ_SA_ = 0.040, 90% CI = 0.018–0.47) corresponding to female effective population sizes of 9,406 (90% CI = 4,362–16,804) for Central America and 111,787 (90% CI = 51,910–1,300,445) for South America. These estimates are similar to, but higher than, the total effective population size of female harpy eagles estimated in MDIV (2.78, 2.52–3.04 95% CI; Figure 3), which corresponds to 51,544 female harpy eagles (27,145–89,728 95% CI). Parameter estimates from coalescent-based analyses in MDIV produced bell-shaped curves with the exception of *T*, which peaked and approached but did not converge to zero in the upper portion of its distribution (Figure 3) and thus we did not use it to calculate estimates of divergence time.

There are no rigorous census estimates for harpy eagles to our knowledge, although Ferguson-Lees and Christie [6] provide a “guess at a five figure population” based on total rainforest habitat area and inter-nest distances of 3–5 km in Panama, Venezuela and Guyana. As seen here, census or point estimates are often smaller than coalescent-based effective population size estimates because a coalescent-based estimate is a reflection of the long-term effective size of the population over a longer time-scale than a contemporary point estimate of population size [e.g.], [51], [52],[53]. Given the lack of certainty in the census “guess” by Ferguson-Lees and Christie [6] and the wide confidence intervals around our long-term female effective population size estimates, further data are needed to fully evaluate harpy eagle population sizes.

The degree of genetic isolation observed with significant *F*_ST_ values was further investigated by estimating levels of migration with the coalescent-based analyses in the programs Migrate and MDIV. Likelihood ratio tests in the Migrate analyses rejected the null hypothesis of symmetric migration (p<0.001). Higher rates of female gene flow from Central America into South America (*m_CA_*  = 694.27, 95% CI 341.8–1306.9; *m_SA_* = 0.000002, 95% CI 0.000001–.0050) were estimated by the mtDNA data. With MDIV, estimates of migration were relatively low, 1.08 (0.83–1.33 95% CI) but significantly different from zero (we are unable to estimate migration rates for Central and South America separately with MDIV because the model assumes symmetric migration between populations).

### Conservation implications

For species in intermediate stages of divergence it is often difficult to differentiate between the alternative possibilities of contemporary gene flow and recent isolation with retention of ancestral polymorphism due to incomplete lineage sorting. The data presented here are consistent with a pattern of intermediate polyphyly (i.e. neotypy [54]) in which restriction of gene flow is evident from coalescent analyses, the haplotype network and significant *F*_ST_ values, however reciprocally monophyletic lineages do not correspond to geographical locations in maximum likelihood analyses. Captive breeding programs that wish to breed eagles in a manner consistent with historical patterns of gene flow may want to limit the introduction of South American mitochondrial haplotypes into Central America, although the recovery of three shared mitochondrial haplotypes between these broad regions suggests that total isolation of captive breeding stocks by geographic origin may not be necessary. Furthermore, it would be best to evaluate these trends with nuclear data and additional sampling of individuals before modifying existing captive-breeding and release programs.

To better interpret the amount of overall genetic variability found for harpy eagles, it is useful to compare these results with patterns of diversity found in related species [55]. With respect to mitochondrial control region sequence data published for nine other taxa in the family Accipitridae, we found the second highest levels of haplotype and nucleotide diversities for harpy eagles (Table 3).

**Table 3:** Genetic diversity of the control region as reported in published studies of Accipitridae taxa.

Species (n = sample size)	IUCN^1^ status	Control region domain (bp)	Number of variable sites (%)	Number of haplotypes	Haplotype diversity, *h* (SD)	Nucleotide diversity, π (SD)
*Aquila adalbarti^6^* (n = 60)	VU	I (345)	2 (0.6)	3	0.321 (0.073)	0.00098 (0.00024)
*Aquila heliaca^6^* (n = 34)	VU	I (345)	8 (2.3)	7	0.779 (0.042)	0.00548 (0.00068)
*Buteo galapagoensis* 2 (n = 122)	VU	I (415)	5 (1.2)	5	0.625 (0.025)	0.0019
***Harpia harpyja*** ** (n = 66)**	**NT**	**I (400)**	**21 (5.3)**	**23**	**0.906 (0.020)**	**0.00763 (0.0045)**
*Milvus milvus* ^7^ (n = 105)	NT	I (357)	10 (2.8)	10	0.610	0.0032
*Buteo swainsoni* 2 (n = 18)	LC	I (415)	18 (4.3)	12	0.766 (0.081)	0.0059
*Gypaetus barbatus^3^* (n = 172)	LC	I (228)	28 (12.3)	50	0.932 (0.012)	0.0292 (0.0153)
*Haliaeetus leucogaster^4^* (n = 128)	LC	I (390) and II (163)	15 (2.7)	15	0.3497 (0.05447)	0.000806 (0.0008)
*Hieraaetus fasciatus^5^* (n = 72)	LC	I (253)	3 (1.2)	4	0.542 (0.046)	0.0024 (0.0017)
*Haliaeetus albicilla^8^* (n = 228)	LC	I and II (500)	12 (2.4)	13	0.746	0.00680 (0.00012)

1 World conservation union red list status, vulnerable (VU), near-threatened (NT), least concern [LC, 16].

2 [89].

3 [90].

4 [91].

5 [92].

6 [93].

7 [94].

8 [95].

Such high levels of genetic diversity were not expected because recent habitat loss and evidence for local extinctions of harpy eagles throughout much of Central and South America [10] might yield population declines and reduced levels of genetic diversity [56], [but see 57], [58]. However, our results should not be interpreted as evidence against recent population declines for harpy eagles. Long-lived species such as the white-tailed eagle [*Haliaeetus albicilla*, 59] have been shown to maintain high levels of genetic diversity even after experiencing relatively recent (<100 years) population bottlenecks. Harpy eagles are a long-lived species, with an estimated longevity of 35 years or more in the wild, and this characteristic may have buffered against immediate loss of mitochondrial genetic diversity associated with recent habitat loss and human persecution. For the white-tailed eagle, the focus on the preservation of multiple local populations, as opposed to one large central population, was suggested by Hailer *et al.* [59] to help maintain high levels of genetic diversity during the recent recovery from a severe population decline. In this case, the recovery of a long-lived bird of prey was achieved due to timely conservation efforts focused on the geographic distribution of genetic diversity. A similar approach has been followed with the oriental white-backed vulture (*Gyps bengalensis*) in southeast Asia where the species has experienced >95% decline in the past fifteen years with a minimal decrease in levels of neutral genetic diversity observed prior to the initiation of a large-scale captive breeding program using birds originating over a wide geographic distribution in Pakistan, India and Nepal [60].

### Conclusions

Our analyses indicate that recent population declines for harpy eagles have not yet caused a reduction in the levels of mitochondrial genetic diversity below those reported for other Accipitridae populations. Future studies should compare these results with estimates from nuclear DNA (perhaps using recently developed microsatellite loci [61]) to see if the restriction in mitochondrial gene flow is also present in the nuclear genome indicating that harpy eagles may be locally inbred. Evidence for geographic differentiation within South America and also between Central and South America support a conservation strategy that focuses on maintaining diverse local populations rather than any single extant population, in order to preserve the maximal level of genetic diversity. Given the important role that harpy eagles serve as a top predator in helping to regulate populations of species at multiple trophic levels, local conservation actions on behalf of the harpy eagle should also help to preserve ecosystem function.

## Materials and Methods

### Samples

Harpy eagle samples were collected from all South American and most Central American countries in the current range of the species (see Supplement, Table S1). The majority of samples are from specimens collected after 1960; however, ten sampled specimens were collected between 1902 and 1938 and one sample was collected in 1868. The samples obtained from museum collections were used to represent geographic areas where harpy eagles have been extirpated (e.g., Mexico) or from countries where the current export of tissue samples is difficult (e.g., Brazil). Because of the larger area of intact rain forest habitat in Panama as compared to other Central American countries and the availability of samples from collaborators, Panamanian samples dominated the Central American dataset (i.e. 26 of 32 samples). The crested eagle, the sister species to the harpy eagle [7], was included as an outgroup for the phylogenetic analyses.

### DNA sequences

DNA was extracted from blood, feathers, and organ tissues using a DNeasy Extraction Kit (QIAGEN, Inc.), with 30 µl of 100 ng/ml dithiothreitol (DTT) added to the extraction buffer when working with feathers. DNA extraction from museum toe pads was performed as described in Lerner and Mindell [7] and conducted in a facility reserved for ancient DNA work at the University of Michigan Museum of Zoology using protocols developed for ancient DNAs including negative extraction and blank amplification controls [62], [63].

We designed four primers to amplify 417 bp of domain I of the mitochondrial control region (Table S2). PCR amplification was performed using Platinum Taq polymerase (Invitrogen). Amplification products were purified on 1.5% low-melting point agarose gels, excised and recovered with a Gel Extraction Kit (QIAGEN, Inc.). PCR products were sequenced on an ABI 3730 automated sequencer. Sequences were aligned by eye in BioEdit Sequence Alignment Editor [64], and unique haplotype sequences were deposited in GenBank (accession numbers GQ917189–GQ917211).

### Analyses

Samples were grouped by geographic regions (see Table 2) to test for possible effects of barriers to gene flow such as mountains and discontinuities of lowland forest. The Andean mountains bisect the rainforest habitat of Panama and western Colombia from the Amazon basin forming a barrier known to limit gene flow in a variety of organisms [e.g. passerine birds], [65], [butterflies], [66], howler monkeys, [67], rainforest trees, [68]. A high degree of natal philopatry and a lack of sightings of soaring harpy eagles over or between rain forest habitats could correspond to local phylogeographic structure, however these tendencies could be countered by rare long-distance dispersal [e.g. harpy eagles released in Belize have traveled over 300 km from the release site, 69]. Therefore, few geographic features may practically act as long-term barriers to gene flow for this species. To investigate regional gene flow, we identified two major regions (1) Central America (including the Darien of Panama and western Colombia), and (2) South America. Within the Central American region we grouped individuals from Mexico, Nicaragua and Costa Rica separately from Panamanian birds based on the lack of continuity of lowland tropical forest between these areas and evidence of corresponding geographic structure in other organisms, including other top predators utilizing a similar prey base [12], [13], [68]. Within South America we defined a northeastern subgroup (Guyana, Surinam and Venezuela), a western subgroup (Ecuador, Peru, eastern Colombia) and a southern subgroup (Brazil and Bolivia) based on proximity of collection sites and genetic divergence amongst these areas in other Neotropical organisms (see above).

The level of genetic diversity within regions and subgroups was estimated by calculating the number of haplotypes, haplotype diversity (*h*) and nucleotide diversity (*π*) using the program Arlequin v. 3.0.1 [70]. To visualize the relationships among haplotypes we inferred a median-joining network [71] using the program Network, v. 4.5.1.0 (available at www.fluxus-engineering.com) with varying genetic distance parameter epsilon (*e* = 0, 10, and 20), and equal weights for transitions and transversions. The median-joining approach followed by the maximum parsimony (MP) option returns a network that corresponds most closely to the strict consensus of maximum parsimony trees found in phylogenetic analyses [72].

Potential for monophyly between harpy eagle haplotypes in Central and South America was assessed using a maximum likelihood analysis in PAUP*. A heuristic search with 10 random addition sequence replicates and 100 bootstrap replicates under the HKY model of sequence evolution [73] selected using ModelTest [74] was performed with and without constraining monophyly of Central American and South American individuals. The significance of the difference in likelihood scores was evaluated using a parametric bootstrap in which 1000 data matrices of 400 bases (the size of the final dataset excluded sites where any individuals had unknown bases) were simulated using the HKY model in Mesquite [75]. Each simulated dataset was subjected to a maximum likelihood analysis as described above, with and without monophyly constraints. The difference in likelihood scores between these runs comprised the null distribution against which the likelihood value from the harpy eagle dataset was tested.

The degree of population differentiation among regions was estimated with *F_ST_* using Tamura-Nei corrected distances between sequences. Partitioning of genetic variance among geographic regions, among subgroups within regions and within subgroups was determined with hierarchical analyses of molecular variance using haplotype frequencies [AMOVA, 76], and significance was determined based on 16002 non-parametric permutations. AMOVA and *F*_ST_ calculations were performed in Arlequin v. 3.0.1 [70].

Demographic histories of harpy eagles in Central and South America were evaluated with three approaches: standard quantitative test statistics, mismatch distributions and coalescent-based estimations. To test for genetic signatures of recent population size changes, Fu's test of neutrality [Fs, 42], Tajima's *D* [77] and Fu and Li's *F* and *D_F_* test statistics [78] were compared among regions and subgroups. Both Fu's *F_S_* and Tajima's *D* use the infinite site-model without recombination to test for departures from selective neutrality and population equilibrium for intraspecific data. Fu's *F_S_* uses information from the haplotype distribution and is particularly sensitive to population demographic expansion where low *F_S_* values indicate an excess of single substitutions usually due to expansion [42]. Tajima's *D* uses the average number of pairwise differences and number of segregating sites in the intraspecific DNA sequence to test for departure from neutral expectations, generally assuming negative values in populations that have experienced size changes, or for sequences that have undergone selection. In populations that have undergone recent bottlenecks or have genetic substructure, values for Tajima's *D* are typically positive [79]. Fu and Li's *F* and *D_F_* compare mutations observed within a population to an outgroup sequence, using information from the number of recent mutations as evidence of recent expansion. Negative values of Fu and Li's *F* and *D_F_* indicate an excess of rare alleles and recent mutations that are consistent with an increase in population size or recent selective sweep, whereas positive values reflect an excess of alleles at intermediate frequency that can result from population bottlenecks or balancing selection [78]. Fu's *F_S_* and Tajima's *D* were calculated in Arlequin v. 3.0.1with 1000 random permutations and Fu and Li's *F* and *D_F_* were estimated in DNAsp [80].

The demographic history of each region was investigated by comparing the shape of their respective mismatch distributions calculated in Arlequin v. 3.0.1 to that expected in stationary and expanding populations. For samples drawn from populations that are at demographic equilibrium, mismatch distributions are usually multimodal [81], whereas unimodal distributions are typically associated with populations that have experienced recent expansions [45]. The distribution of the sum of squared differences (SSD) between the observed mismatch distribution for each region and a mismatch distribution estimated under a model of population expansion is used as a test statistic where a significant SSD value indicates departure from a model of sudden population expansion [82]. To estimate the time of expansion (*t*) we converted the parameter τ, estimated from the mismatch distribution, using the equation τ = 2*μt* [83]. nfidence intervals for τwere calculated using a parametric bootstrap approach [82].

The migration rate between regions and relative effective population sizes (*θ* = *N*_efμ_, where *N*_ef_ is the female effective population size and μ is the divergence rate per locus per year) were estimated with MIGRATE [v. 2.1], [84], [85]. Estimates of *θ* generated from default settings were used as initial starting points for final runs. Three final runs were conducted to verify convergence upon similar values using the following parameters: 10 short chains of 100 000 steps and two long chains of 20 000 000 steps with sampling every 100 steps and a burnin of 200 000 steps. Likelihood ratio tests were performed in each final run to evaluate the support for symmetric versus asymmetric migration.

To evaluate the differing scenarios of recurrent gene flow and ancestral polymorphism we used two coalescent-based methods that simultaneously estimate gene flow and divergence times. Estimates of the female effective population sizes (*θΤ* = 2*N*_ef_μ, where *N*_ef_ is the female effective population size and μ is the divergence rate per locus per year), migration between the regions (*M* = 2*N*_ef_μ), time since divergence (*T* = *t*/2*N*_ef_ where *t* is the generation time) and time to most recent common ancestor (TMRCA = *t*μ) were estimated using a Bayesian likelihood approach with the HKY finite sites model in the program MDIV [86]. We conducted three independent runs using different random number seeds to evaluate convergence upon similar values of the modes in posterior distributions. Upper bounds for *M*, *θΤ*, and *T* were set to ten. The posterior distribution of *T* approached but did not reach zero in the upper portion of the distribution, so additional analyses were performed with an upper bound of 20. The posterior distribution for runs with this larger prior remained level rather than converging upon zero, so runs using the smaller prior are reported here. The length of the Markov chain was set to 2.5 million generations with a burnin of 500,000 generations. Posterior distributions for the parameters were plotted and the mode of the posterior distribution was selected as the best estimate with the exception of the parameter *T*, where the point with the highest likelihood value was used.

To convert parameter estimates generated by MIGRATE and MDIV to biologically informative values, an estimate of the neutral mutation rate per generation is needed for the control region. Average time between breeding attempts is 3–5 years, we used 4 years (Jose de J. Vargas, pers. comm.). A mutation rate has not been calibrated for any Accipitridae species, so we used a range of mutation rates originally calculated for the control region in grouse [4.54–12.54% [average 7.23%] divergence per million years, 87]which is similar to that found for the most variable part of the control region in diving ducks [8.8%, 88]. When converting maximum likelihood estimates and modes of parameters we used the average mutation rate of 7.23% divergence per million years. To incorporate the effect of uncertainty around the mutation rate, we used the upper and lower estimates of the mutation rate (4.54–12.54%) to calculate wider credibility intervals (CI) than if we had simply used the average mutation rate. We also applied this method to our estimates of τ.

## Supporting Information

Table S1 Sample information for harpy eagles (Harpia harpyja) and one outgroup (Morphnus guianensis) analyzed in this study
(0.10 MB DOC)

Table S2 Primer sequences used for the amplification of the mitochondrial control region in harpy eagles
(0.03 MB DOC)
