# Supplementary material for: It's not too Late for the Harpy Eagle (Harpia harpyja): High Levels Of Genetic Diversity and Differentiation Can Fuel Conservation Programs
Source: PLoS One. 2009 Oct 5;4(10):e7336. doi: 10.1371/journal.pone.0007336 (PMC2752114; doi:10.1371/journal.pone.0007336)
Supplement: Table S1 — Sample information for harpy eagles (Harpia harpyja) and one outgroup (Morphnus guianensis) analyzed in this study (0.10 MB DOC) [file pone.0007336.s001.doc]

| **Source name^1^ and**  **Cat. No.** | Collection **Date** | Collection Locality | **Tissue Type** | **Haplotype** |
| --- | --- | --- | --- | --- |
| Bell-07 | 1994 | Venezuela | Liver | Hh14 |
| WFVZ 10471 | 1962 | Mexico | Feather | Hh11 |
| TPF Freedom | 1997 | Unknown | Blood | Hh12 |
| TPF 94SD | 1997 | Colombia | Blood | Hh16 |
| TPF 095^2^ | 1997 | Ecuador | Blood | Hh16 |
| TPF GBGrey | 1997 | Venezuela | Blood | Hh14 |
| TPF FRBL | 1997 | Panama | Blood | Hh13 |
| TPF CHEY | 1997 | Unknown | Blood | Hh19 |
| TPF CRAWL | 1997 | Venezuela | Blood | Hh14 |
| TPF COCA | 1997 | Ecuador | Blood | Hh15 |
| TPF OLIVA | 1997 | Venezuela | Blood | Hh20 |
| TPF OLAFA | 1997 | Ecuador | Blood | Hh16 |
| TPF MilZoo | 1997 | Ecuador | Blood | Hh17 |
| WFVZ 001 | 1999 | Guyana | Feather | Hh21 |
| WFVZ 002 | 1999 | Guyana | Feather | Hh20 |
| LSUMZ 111050 | 1982 | Peru | Feather | Hh22 |
| TPF HE-021 | 2004 | Panama | Blood | Hh16 |
| TPF 008 | 2004 | Panama | Blood | Hh16 |
| TPF HE-018 | 2003 | Panama | Blood | Hh16 |
| UMMZ 239465 | 2003 | Panama | Blood | Hh11 |
| TPF 020 | 2004 | Panama | Blood | Hh1 |
| TPF HE-015 | 2004 | Panama | Toepad | Hh20 |
| TPF HE-016 | 2003 | Panama | Toepad | Hh11 |
| TPF HE-007 | 2004 | Panama | Feather | Hh20 |
| TPF HE-01 | 2004 | Panama | Feather | Hh11 |
| TPF HE-017 | 2004 | Panama | Feather | Hh11 |
| TPF HE-014 | 2004 | Panama | Feather | Hh16 |
| TPF HE-010 | 2004 | Panama | Feather | Hh2 |
| TPF HE-001 | 2003 | Panama | Feather | Hh16 |
| TPF CRE-005 | 2002 | Panama | Feather | Hh11 |
| TPF HE-013 | 2004 | Panama | Feather | Hh1 |
| TPF HE-012 | 2004 | Panama | Feather | Hh16 |
| TPF HE-006 | 2003 | Panama | Feather | Hh11 |
| TPF HE-003 | 2002 | Panama | Feather | Hh11 |
| TPF HE-008 | 2004 | Panama | Feather | Hh16 |
| TPF HE-004 | unknown | Panama | Feather | Hh2 |
| TPF HE-002 | 2002 | Panama | Feather | Hh11 |
| TPF HE-009 | 2003 | Panama | Feather | Hh20 |
| UMMZ 239466 | 2005 | Panama | Tissue | Hh14 |
| UMMZ BD-8225 | 2005 | Panama | Tissue | Hh20 |
| UMMZ 239471 | 2005 | Panama | Tissue | Hh16 |
| UMMZ PAN-01 | 2003 | Ecuador | Feather | Hh20 |
| SDZ 402158 | 2004 | Unknown | Feather | Hh16 |
| SB | 2003 | Guyana | Feather | Hh7 |
| KUNH 24802 | unknown | Mexico | Toepad | Hh11 |
| FMNH 260141 | 1964 | Surinam | Toepad | Hh4 |
| FMNH 264326 | 1965 | Surinam | Toepad | Hh4 |
| FMNH 371026 | 1977 | Ecuador | Toepad | Hh16 |
| FMNH 104888 | 1938 | Guyana | Stomach contents | Hh4 |
| FMNH 32150 | unknown | Guyana | Toepad | Hh5 |
| USNM 54224 | 1868 | Mexico | Toepad | Hh11 |
| USNM 193559 | 1902 | Nicaragua | Toepad | Hh11 |
| USNM 253473 | 1917 | Brazil | Toepad | Hh8 |
| JMM-A-3224 | 1960 | Guyana | Toepad | Hh6 |
| MCZ 58503 |  | Costa Rica | Toepad | Hh13 |
| ROM 94251 | 1963 | Guyana | Toepad | Hh22 |
| LSUMZ 31239 | 1963 | Peru | Toepad | Hh18 |
| LSUMZ 35120 | 1964 | Peru | Feather | Hh19 |
| LSUMZ B-51351 | 1963 | Peru | Toepad | Hh18 |
| LSUMZ B-51352 | 1963 | Peru | Toepad | Hh10 |
| LSUMZ 51268 | 1946 | Bolivia | Toepad | Hh19 |
| AMNH 102432 | 1911 | Nicaragua | Toepad | Hh11 |
| AMNH 238836 | 1932 | Peru | Toepad | Hh3 |
| AMNH 406859 | 1931 | Peru | Toepad | Hh9 |
| AMNH 429102 | 1935 | Brazil | Toepad | Hh12 |
| TPF CUBL.2IH | 1997 | Ecuador | Feather | Hh16 |
| AMNH 272336 | 1932 | Venezuela | Toepad | Hh20 |
| HUA, *Morphnus guianensis* | 2003 | Peru | Blood | n/a |

^1^Bell Museum of Natural History (BELL), Western Foundation of Vertebrate Zoology (WFVZ), Louisiana State University (LSUMZ), The Peregrine Fund (TPF), Royal Ontario Museum (ROM), Museum of Comparative Zoology (MCZ), American Museum of Natural History (AMNH), Joseph Moore Museum (JMM), El Huayco, Peru (HUA), San Diego Zoo (SDZ), Sue Boinski (SB), Western Foundation of Vertebrate Zoology (WFVZ), University of Michigan Museum of Zoology (UMMZ), National Museum of Natural History (USNM), Field Museum of Natural History (FMNH)

^2^sibling of HH-41, excluded from all analyses
