# Supplementary material for: It's not too Late for the Harpy Eagle (Harpia harpyja): High Levels Of Genetic Diversity and Differentiation Can Fuel Conservation Programs
Source: PLoS One. 2009 Oct 5;4(10):e7336. doi: 10.1371/journal.pone.0007336 (PMC2752114; doi:10.1371/journal.pone.0007336)
Supplement: Table S2 — Primer sequences used for the amplification of the mitochondrial control region in harpy eagles (0.03 MB DOC) [file pone.0007336.s002.doc]

| Primer ID | Sequence (5’-3’) |
| --- | --- |
| LDL-1 | CCCATTATCATGCACTATTCTAGG |
| HDL-1 | GAGCAAGGTCGTAGGACTAACC |
| HDL-3 | ATAACCTGGTCCGACAYACG |
| LDL-3 | CGGATATTCTTGGGGACAAA |
